# SnapHiC: a computational pipeline to identify chromatin loops from single-cell Hi-C data

**DOI:** 10.1038/s41592-021-01231-2

**Published:** 2021-08-26

**Authors:** Miao Yu, Armen Abnousi, Yanxiao Zhang, Guoqiang Li, Lindsay Lee, Ziyin Chen, Rongxin Fang, Taylor M. Lagler, Yuchen Yang, Jia Wen, Quan Sun, Yun Li, Bing Ren, Ming Hu

**Affiliations:** 1 State Key Laboratory of Genetic Engineering, School of Life Sciences, Fudan University, Shanghai, China; 2 Ludwig Institute for Cancer Research, La Jolla, CA USA; 3 Department of Quantitative Health Sciences, Lerner Research Institute, Cleveland Clinic Foundation, Cleveland, OH USA; 4 Howard Hughes Medical Institute, Department of Chemistry and Chemical Biology, Harvard University, Cambridge, MA USA; 5 Department of Biostatistics, University of North Carolina, Chapel Hill, NC USA; 6 Department of Pathology and Laboratory Medicine, University of North Carolina, Chapel Hill, NC USA; 7 McAllister Heart Institute, University of North Carolina, Chapel Hill, NC USA; 8 Department of Genetics, University of North Carolina, Chapel Hill, NC USA; 9 Department of Computer Science, University of North Carolina, Chapel Hill, NC USA; 10 Center for Epigenomics, Department of Cellular and Molecular Medicine, University of California, San Diego, La Jolla, CA USA

**Keywords:** Computational models, Chromatin

## Abstract

Single-cell Hi-C (scHi-C) analysis has been increasingly used to map chromatin architecture in diverse tissue contexts, but computational tools to define chromatin loops at high resolution from scHi-C data are still lacking. Here, we describe Single-Nucleus Analysis Pipeline for Hi-C (SnapHiC), a method that can identify chromatin loops at high resolution and accuracy from scHi-C data. Using scHi-C data from 742 mouse embryonic stem cells, we benchmark SnapHiC against a number of computational tools developed for mapping chromatin loops and interactions from bulk Hi-C. We further demonstrate its use by analyzing single-nucleus methyl-3C-seq data from 2,869 human prefrontal cortical cells, which uncovers cell type-specific chromatin loops and predicts putative target genes for noncoding sequence variants associated with neuropsychiatric disorders. Our results indicate that SnapHiC could facilitate the analysis of cell type-specific chromatin architecture and gene regulatory programs in complex tissues.

## Abstract (web summary)

SnapHiC offers a computational tool for improving detection of chromatin loops from single-cell Hi-C data.

## Main

Single-cell Hi-C (scHi-C) technologies have been developed to map chromatin architecture in individual cells, enabling the measure of spatial proximity between transcriptional regulatory elements in a cell type-specific manner^1–3^. However, due to the lack of tools tailored for scHi-C data, identifying loops from scHi-C data mainly relies on applying methods developed for bulk Hi-C^4,5^ to the aggregated scHi-C data of the same cell type. Due to the extreme sparsity of scHi-C data, such a strategy would require a large number of cells (>500–1,000), which is both cost prohibitive and impractical for rare cell types. To overcome these issues, we developed Single-Nucleus Analysis Pipeline for Hi-C (SnapHiC), a computational framework customized for scHi-C data to identify chromatin loops at high resolution and accuracy from a small number of cells.

**Fig. 1:**
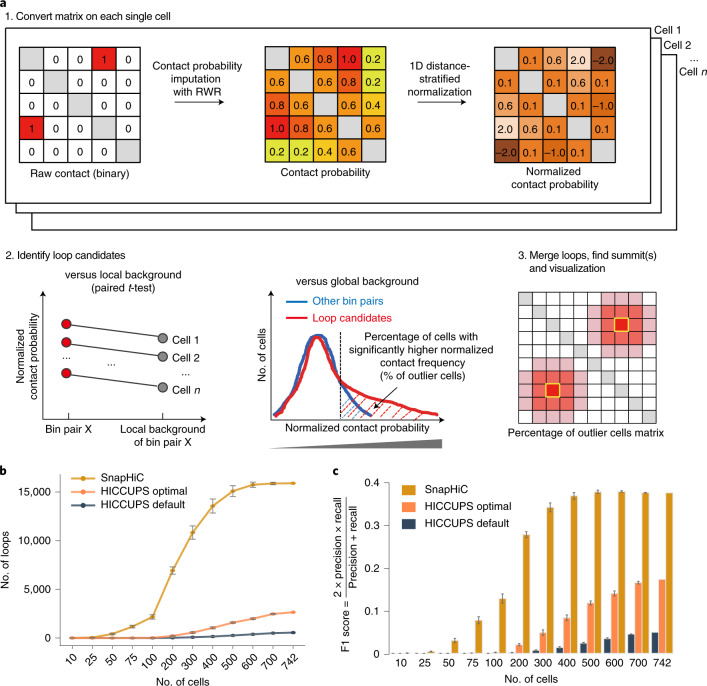
SnapHiC reveals chromatin loops at high resolution and accuracy. **a**, Overview of the SnapHiC workflow. **b**, The number of chromatin loops at 10-kb resolution identified by SnapHiC and HiCCUPS (with default or optimal parameters) from different numbers of mES cells. **c**, F1 score of SnapHiC- and HiCCUPS-identified loops (with default or optimal parameters) from different numbers of mES cells. In **b**,**c**, the dots and the error bars represent the mean values and the standard deviations calculated across six randomly sampled subsets, respectively. Source data

SnapHiC (Fig. 1a) first imputes intrachromosomal contact probability between pairs of 10-kilobase (kb) bins in each cell with the random walk with restart (RWR) algorithm^6^. Next, it normalizes the imputed contact probability based on linear genomic distances. SnapHiC then applies paired *t*-test to the matrices of normalized contact probability of all cells to identify candidate bin pairs (or loop candidates) with higher-than-expected contact probability in a population of cells. To minimize false positives, SnapHiC considers a bin pair as a loop candidate only when its normalized contact probability is significantly higher than expected by chance based on both global and local background. Finally, SnapHiC groups the loop candidates into clusters^7^ and identifies the summit(s) within each cluster. In SnapHiC, individual cells are treated as independent datasets instead of being aggregated into pseudo bulk data. Therefore, the variability of contact frequency within the cell population can be estimated to boost the statistical power in loop detection, especially when the number of cells is low.

**Extended Data Fig. 1:**
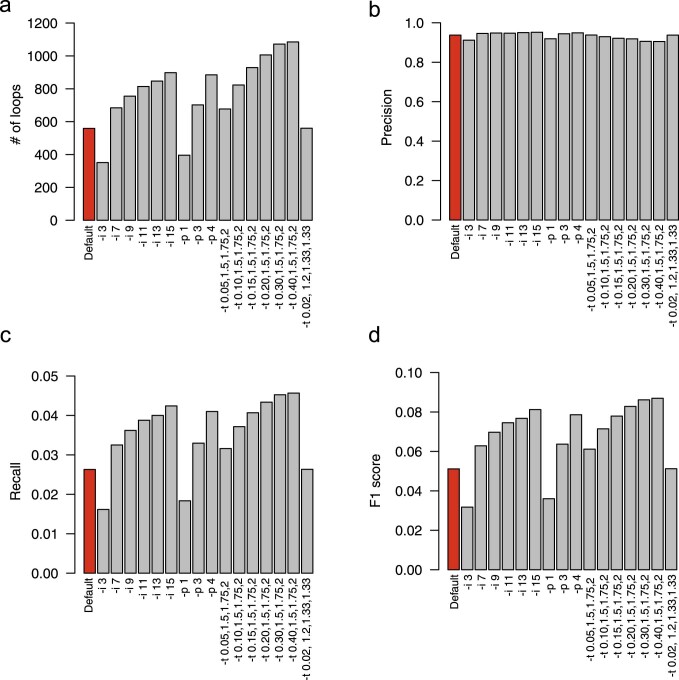
Optimization of HiCCUPS parameters using aggregated scHi-C data of the 742 mES cells. The number of loops within 100Kb to 1 Mb range (**a**), precision rate (**b**), recall rate (**c**) and F1 score (**d**) for HiCCUPS loops running with different parameters. Default parameter: -f .1 -p 2 -i 5 -t 0.02,1.5,1.75,2 -d 20000. Related to Supplementary Note.

**Extended Data Fig. 2:**
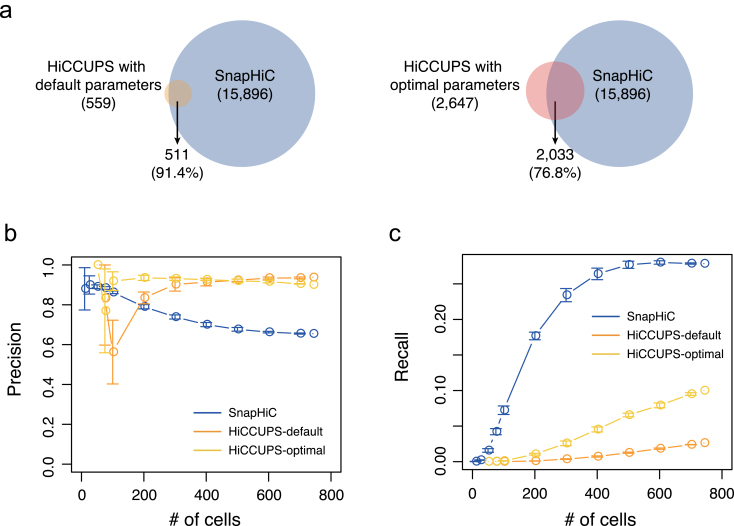
Comparison of SnapHiC- and HiCCUPS-identified loops from mES cells. **a**, Venn diagram of overlaps between SnapHiC- and HiCCUPS-identified loops (with default or optimal parameters) from 742 mES cells. **b**, **c**, Line plots showing the performance of SnapHiC and HiCCUPS applied to different number of mES cells (N=10, 25, 50, 75, 100, 200, 300, 400, 500, 600, 700 and 742). The dots represent the mean values of precision rate (**b**) and recall rate (**c**) across six randomly sampled subsets of mES cells from the 742 cells that passed our quality control (see details in Methods), respectively (except for N=742). The error bar represents the standard deviation calculated across six randomly sampled subsets. These values are also used to calculate the F1 score in Fig. 1c. If the lower bound of confidence interval (mean-sd) is less than 0, it is set as 0. For precision, recall and F1-score, if the upper bound of confidence interval (mean+sd) is greater 1, it is set as 1. Source data

We first benchmarked the performance of SnapHiC against a commonly used loop detection method for bulk Hi-C data, HiCCUPS^4^. We applied SnapHiC to the published scHi-C data^1^ generated from mouse embryonic stem (mES) cells. Besides the full set of 742 cells, we also randomly subsampled 10, 25, 50, 75, 100, 200, 300, 400, 500, 600 and 700 cells from this dataset, and determined 10-kb-resolution intrachromosomal loops within the 100 kb–1 Mb range. For each subsampling, we also pooled the scHi-C data and identified chromatin loops at 10-kb resolution using HiCCUPS with both default and ‘optimal’ parameters for sparse data (Supplementary Note and Extended Data Fig. 1). For each subsampling dataset, SnapHiC found substantially more loops than HiCCUPS, suggesting SnapHiC has a much higher sensitivity than HiCCUPS (Fig. 1b and Supplementary Table 1). Even from 75 cells, SnapHiC identified 1,050–1,420 loops, whereas HiCCUPS found only 0–2 loops with default parameters and 3–10 loops with optimal parameters. Additionally, HiCCUPS-identified loops tended to be a subset of SnapHiC-identified loops (Extended Data Fig. 2a). Moreover, SnapHiC achieved higher reproducibility. From two replication datasets with 371 cells each, reproducibility was 50.8% for SnapHiC versus 38.7% for HiCCUPS with default parameters (paired *t*-test two-sided *P* = 7.86 × 10^−8^), while 50.8% for SnapHiC versus 39.7% for HiCCUPS with optimal parameters (paired *t*-test two-sided *P* = 9.90 × 10^−11^).

**Extended Data Fig. 3:**
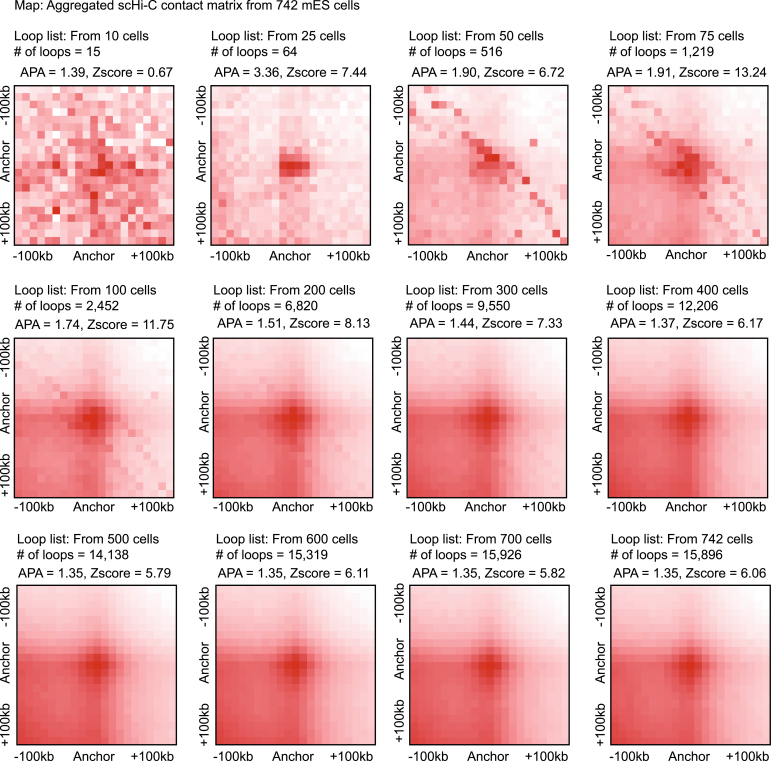
SnapHiC-identified loops from different sub-sampling of mES cells showed significant enrichment over their local backgrounds. Aggregate peak analysis (APA) of SnapHiC-identified loops from different sub-sampling of mES cells examined on aggregated scHi-C contact matrix of 742 cells.

**Extended Data Fig. 4:**
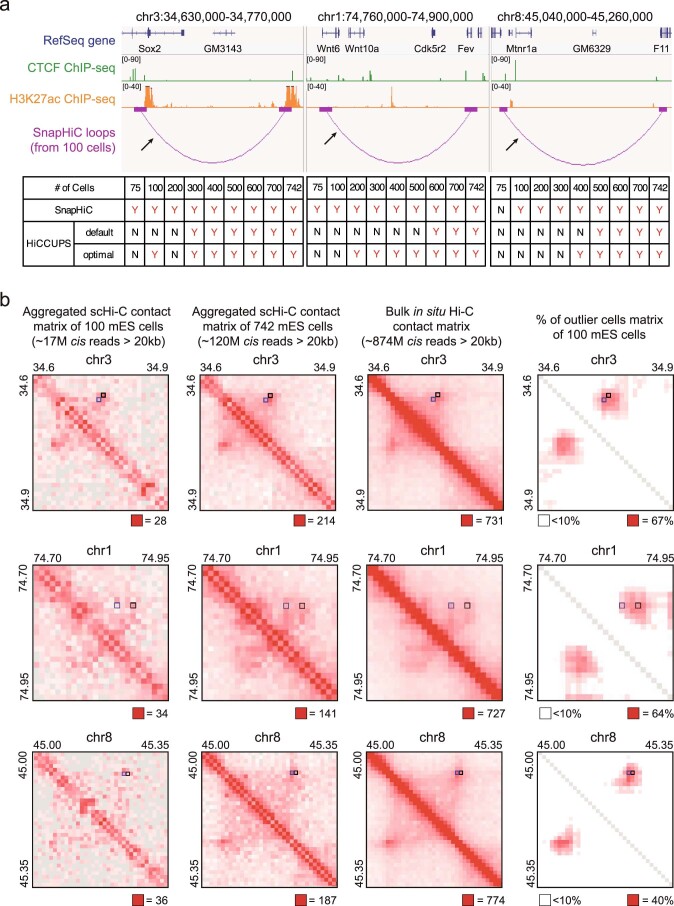
Performance of SnapHiC and HiCCUPS at *Sox2*, *Wnt6* and *Mtnr1a* loci. **a**, (Top) Chromatin loops around *Sox2* (left), *Wnt6* (middle), and *Mtnr1a* (right) gene identified from 100 mES cells using SnapHiC at 10 kb resolution. The black arrow points to the interactions verified in the previous publications^13,14^ with CRISPR/Cas9 deletion or 3C-qPCR. (Bottom) Comparison of the performance of SnapHiC and HiCCUPS (applied on aggregated scHi-C data with default or optimal parameters) from different number of mES cells at these three regions. If the previously verified interaction (black arrow) is recaptured, it is labeled as ‘Y’; otherwise, it is labeled as ‘N’. **b**, From left to right: aggregated scHi-C contact matrix of 100 mES cells, aggregated scHi-C contact matrix of 742 mES cells, bulk in situ Hi-C contact matrix from mES cells (replicate 1 from Bonev et al. study^8^) and % of outlier cells matrix of 100 mES cells at 10 kb resolution; from top to bottom: *Sox2* locus, *Wnt6* locus, and *Mtnr1a* locus. Black squares represent the SnapHiC-identified loops from 100 mES cells, which are shown in (a) as purple arcs. For comparison, the HiCCUPS-identified loops from the deepest available bulk in situ Hi-C data of mES cells (combining all four replicates from Bonev et al. study^8^) are marked as blue squares.

We used the F1 score, the harmonic mean of the precision and recall rates, to evaluate the overall performance of each method. To calculate the F1 score, we combined the chromatin loops identified by HiCCUPS from bulk in situ Hi-C data^8^, with long-range interactions identified by MAPS (model-based analysis of long-range chromatin interactions from PLAC-seq (proximity ligation-assisted ChIP–seq (chromatin immunoprecipitation assays with sequencing)) and HiChIP experiments) from H3K4me3 PLAC-seq data^9^, cohesin^10^ and H3K27ac HiChIP data^11^, all from mES cells. At each subsampling of scHi-C data, SnapHiC consistently attained a greater F1 score than HiCCUPS (Fig. 1c and Extended Data Fig. 2b,c). The reliability of SnapHiC-identified loops was supported by two lines of evidence: (1) significant focal enrichment at anchors of loops identified from at least 25 cells was observed from aggregate peak analysis (APA) plots using aggregated contact matrices of 742 cells (Extended Data Fig. 3) and (2) for SnapHiC-identified loops with CTCF (CCCTC-binding factor) binding on both anchors, there was a clear preference in convergent orientation—ranging from 63.6% to 78.7% when at least 50 cells are used (Supplementary Table 2), as predicted by the loop extrusion model^4,12^. The advantages of SnapHiC were more obvious when the number of cells profiled is limited. As illustrated in Extended Data Fig. 4, SnapHiC detected previously verified long-range interactions at *Sox2*, *Wnt6* and *Mtnr1a* loci^13,14^ from scHi-C data of as few as 75 cells, whereas HiCCUPS required at least 200–600 cells to detect the same loops.

**Extended Data Fig. 5:**
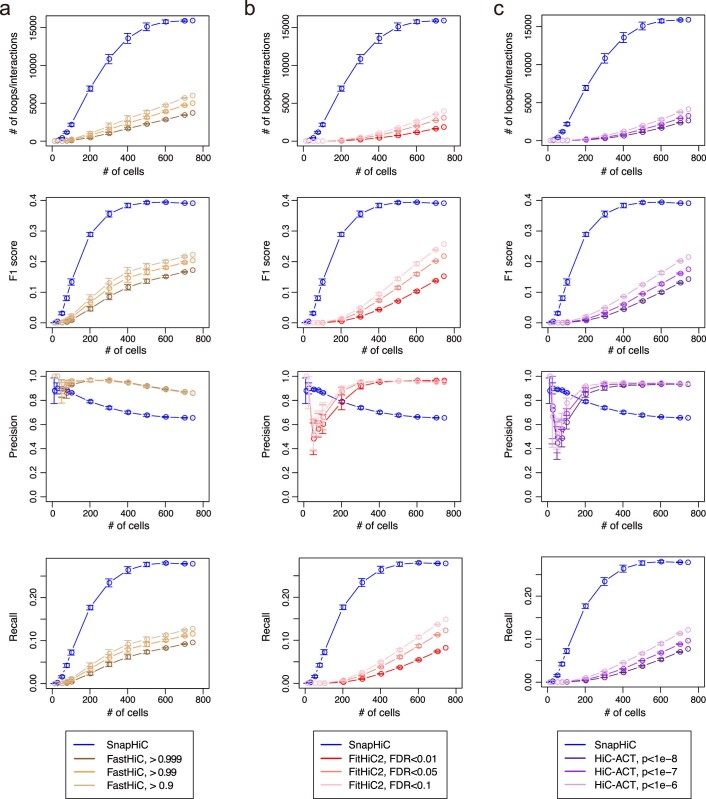
Comparison of the performance of SnapHiC with FastHiC, FitHiC2 and HiC-ACT. The performance of FastHiC **a**, FitHiC2 **b,﻿** and HiC-ACT **c**,﻿ on different numbers of mES cells (N=10, 25, 50, 75, 100, 200, 300, 400, 500, 600, 700 and 742), comparing with SnapHiC. The dots represent the mean values of the numbers of loops/interactions, precision rate, recall rate and F1 score of six randomly sampled subsets of mES cells from the 742 cells that passed our quality control, respectively (except for N=742). The error bar represents the standard deviation calculated across six randomly sampled subsets. For FastHiC (**a**), the posterior probability of being significant interactions >0.999 is commonly used; two more lenient thresholds >0.99 and >0.9 were tested considering the sparsity of single cell Hi-C data. For FitHiC2 (**﻿b**), FDR<0.01 is commonly used; two more lenient thresholds <0.05 and <0.1 were tested considering the sparsity of single cell Hi-C data. For HiC-ACT (**c**), smoothed p-value <1e-8 is commonly used; two more lenient thresholds <1e-7 and <1e-6 were tested considering the sparsity of single cell Hi-C data. The HiC-ACT p-values are calculated based on one-sided aggregated Cauchy test. In **a**, **﻿b** and **﻿c**, if the lower bound of confidence interval (mean-sd) is less than 0, it is set as 0. For precision rate, recall rate and F1-score, if the upper bound of confidence interval (mean+sd) is greater 1, it is set as 1.

**Extended Data Fig. 6:**
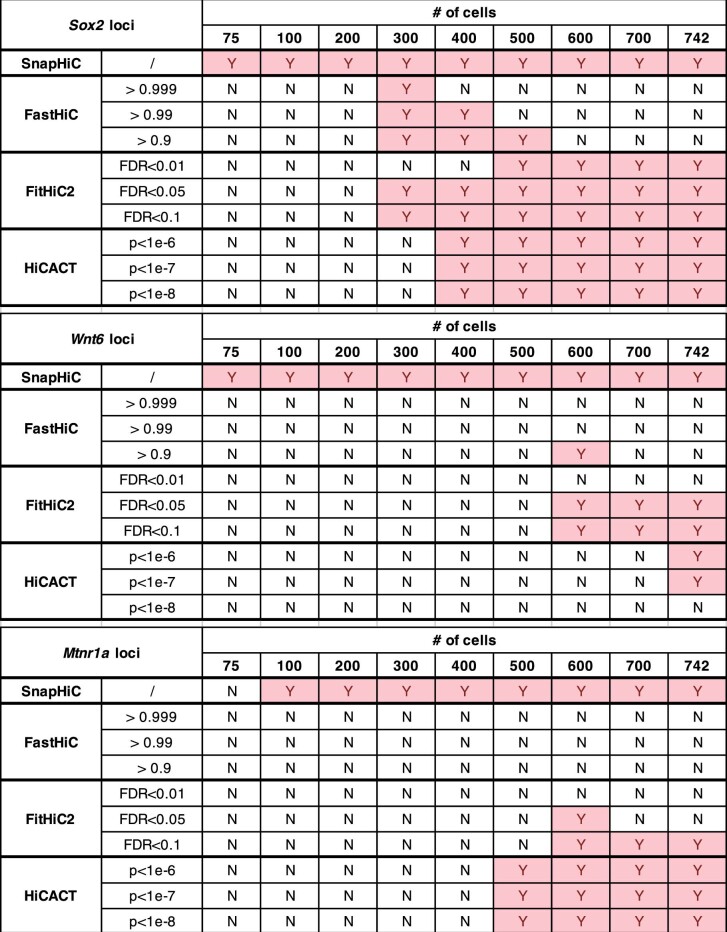
Comparison of the performance of SnapHiC with HiCCUPS, FastHiC, FitHiC2 and HiC-ACT at *Sox2*, *Wnt6* and *Mtnr1a* loci. If the previously verified interaction (black arrow in Extended Data Fig. 4a) is recaptured, it is labeled as ‘Y’; otherwise, it is labeled as ‘N’.

We next compared the performance of SnapHiC with three additional methods designed to identify long-range interactions from bulk Hi-C-FastHiC^15^, FitHiC2 (ref. ^5^) and HiC-ACT^16^ (Supplementary Note). Considering their default thresholds may not be optimal for the sparse scHi-C data, we also tested different thresholds for each method. Results on different numbers of mES cells demonstrated that SnapHiC consistently identified more loops and achieved greater F1 scores than the other methods, with higher recall rates and equivalent or slightly lower precision rates (Extended Data Fig. 5). For the three loci examined above (Extended Data Fig. 4), SnapHiC also detected the known long-range interactions with much fewer cells than the other methods (Extended Data Fig. 6). Taken together, our results suggested that SnapHiC can identify loops from a small number of cells with high sensitivity and accuracy.

**Fig. 2:**
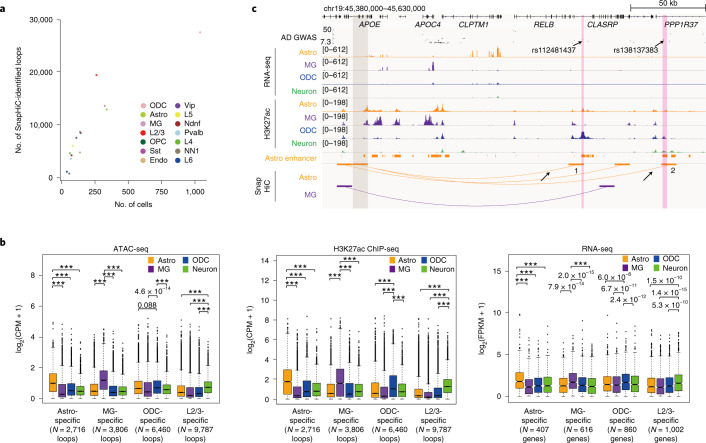
Application of SnapHiC to sn-m3C-seq data from human prefrontal cortex uncovered chromatin loops in diverse brain cell types. **a**, Scatter plot showing the numbers of cells and SnapHiC-identified loops in each of the 14 major cell types identified in human prefrontal cortex in Lee et al.^3^. ODC, oligodendrocyte. Astro, astrocyte. MG, microglia. OPC, oligodendrocyte progenitor cell. Endo, endothelial cell. L2/3, L4, L5 and L6 denote excitatory neuron subtypes located in different cortical layers. Pvalb and Sst, medial ganglionic eminence-derived inhibitory subtypes. Ndnf and Vip, CGE-derived inhibitory subtypes. NN1, nonneuronal cell type 1. **b**, Boxplot of ATAC-seq log_2_(CPM+1) value (left), H3K27ac ChIP–seq log_2_(CPM+1) value (middle) and RNA-seq log_2_(FPKM+1) value (right) at Astro-specific, MG-specific, ODC-specific and L2/3-specific SnapHiC loops. For each set of SnapHiC loops, the values are calculated using ATAC-seq/H3K27ac ChIP–seq/RNA-seq data generated from astrocyte, microglia, oligodendrocytes and neurons, respectively (Methods). ***P < 2.2 × 10^−16^, two-sided *P* values by the paired Wilcoxon signed-rank test. In each box, the upper edge, horizontal center line and lower edge represent the 75th percentile, median and 25th percentile, respectively. The upper whiskers represent the 75th percentile + 1.5× the interquartile range (IQR). The lower whiskers represent the minimum values (0). Data points with a value above the 75th percentile + 1.5× the IQR are outliers and reported as dots. **c**, SnapHiC-identified loops from astrocyte and microglia around gene *APOE*. There is no loop identified in this genomic region from oligodendrocytes or L2/3 excitatory neurons, so no corresponding tracks are shown. Two astrocyte-specific loops linking the *APOE* promoter (highlighted in gray) and the active enhancers in astrocyte (highlighted in pink) containing two Alzheimer’s disease (AD) -associated GWAS SNPs are marked by black arrows. Only *APOE* transcription start site-distal Alzheimer’s disease-associated GWAS SNPs are shown in the figures (residing in the region chr19: 45,440,000–45,630,000). Source data

**Extended Data Fig. 7:**
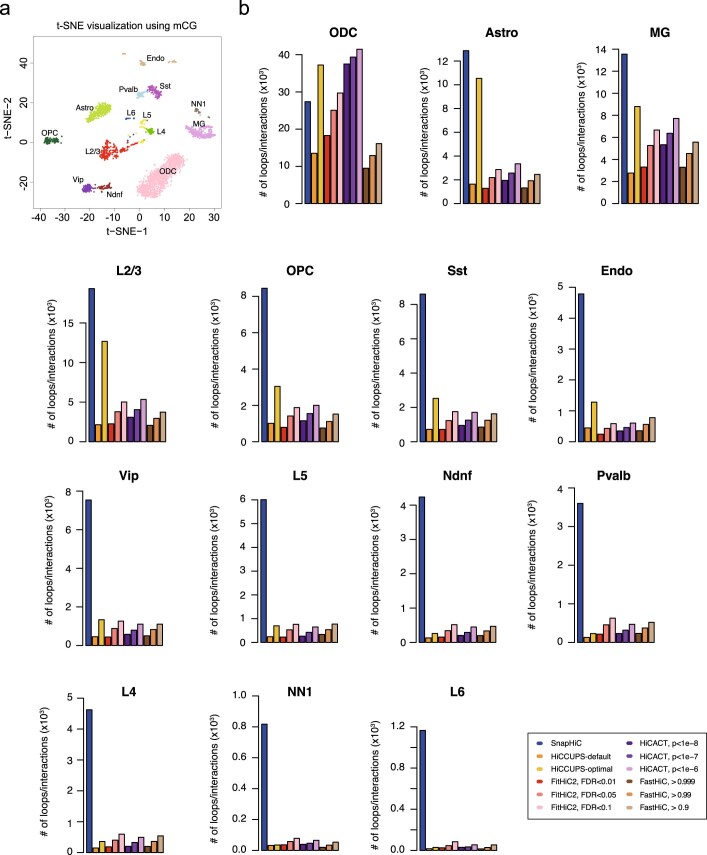
Identification of loops/interactions using sn-m3C-seq data generated from human prefrontal cortex by different methods. **a**, t-SNE visualization of 14 major cell types identified in human prefrontal cortex in Lee et al. study^3^ based on cell-type-specific CG and non-CG methylation patterns. ODC: oligodendrocyte. Astro: astrocyte. MG: microglia. OPC: oligodendrocyte progenitor cell. Endo: endothelial cell. L2/3, L4, L5 and L6: excitatory neuron subtypes located in different cortical layers. Pvalb and Sst: medial ganglionic eminence-derived inhibitory subtypes. Ndnf and Vip: CGE-derived inhibitory subtypes. NN1: non-neuronal cell type 1. **b**, The number of loops/interactions identified from each of the 14 cell types by SnapHiC, HiCCUPS, FitHiC2, FastHiC and HiC-ACT. Source data

**Extended Data Fig. 8:**
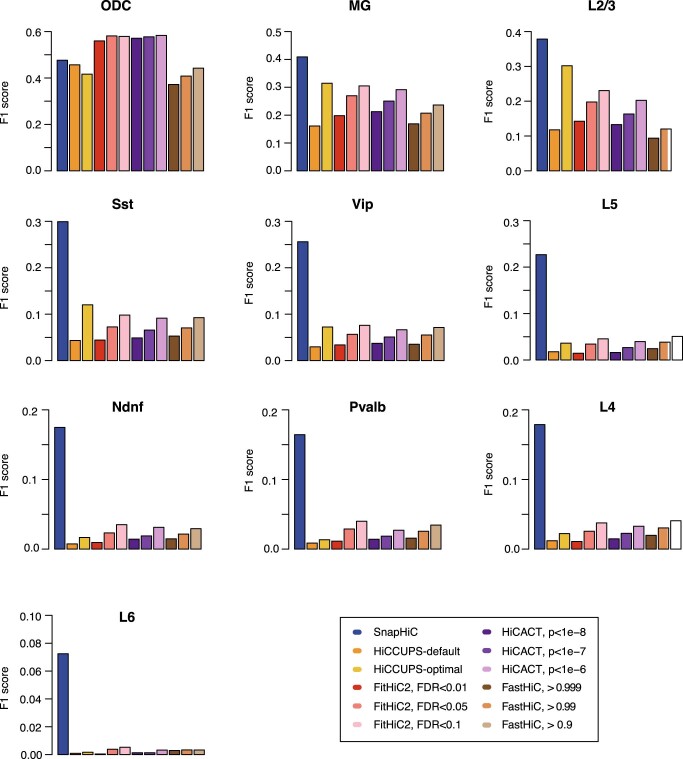
F1 score of SnapHiC-, HiCCUPS-, FitHiC2-, FastHiC- and HiC-ACT-identified loops/interactions for ten cell clusters from human prefrontal cortex. The F1 scores are calculated for oligodendrocytes (ODC), microglia (MG), and eight neuronal subtypes (L2/3, L4, L5, L6, Sst, Vip, Ndnf and Pvalb) using promoter-centered chromatin interactions previously identified from H3K4me3 PLAC-seq data^17^ of purified oligodendrocytes, microglia, astrocytes and neurons, respectively. Source data

To demonstrate the use of SnapHiC on complex tissues, we applied it to the published single-nucleus methyl-3C-seq (sn-m3C-seq) data^3^ generated from human prefrontal cortex, which simultaneously profiled DNA methylome and chromatin organization from the same cells. In this study, 14 major cell types were classified from single-cell methylome data based on cell type-specific CG and non-CG methylation patterns (Extended Data Fig. 7a). We applied SnapHiC to the Hi-C component of each cell within the 14 cell clusters, and identified roughly 817–27,379 loops at 10-kb resolution (Fig. 2a). Consistent with our observation on mES cells, SnapHiC identified more chromatin loops than tools developed for bulk Hi-C (Extended Data Fig. 7b) and yielded the highest F1 score on all cell types except for the oligodendrocytes (Extended Data Fig. 8 and Supplementary Table 3), which had comparable sequencing depth to routine bulk Hi-C data after aggregating (roughly 278 million intrachromosomal reads >20 kb, 1,038 cells).

**Extended Data Fig. 9:**
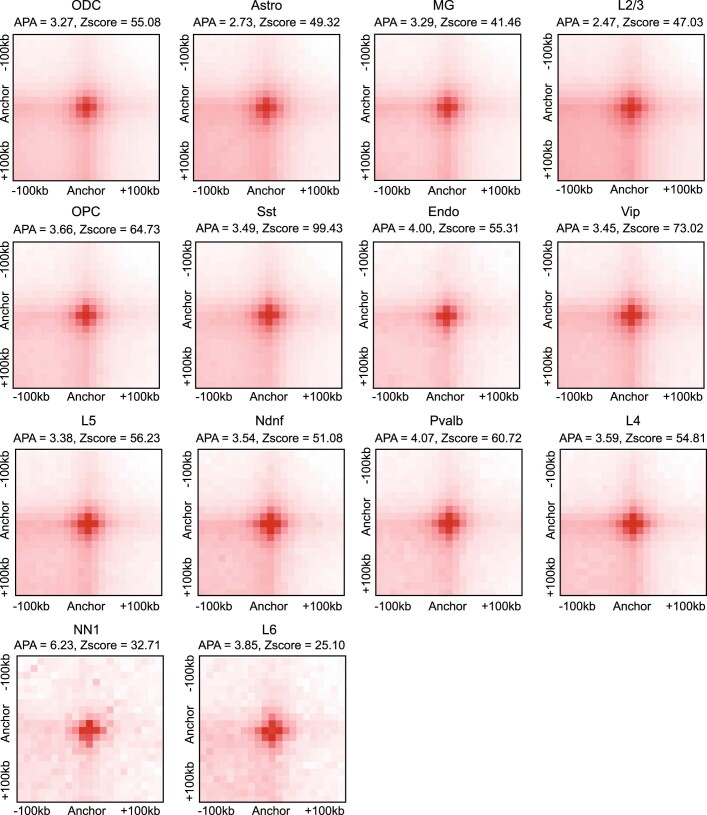
SnapHiC-identified loops from each of the 14 cell clusters identified from sn-m3C-seq data of the human prefrontal cortex show significant enrichment over their local background. Aggregate peak analysis (APA) of SnapHiC-identified loops for each of the 14 cell types demonstrated in Extended Data Fig. 7a examined on the aggregated contact matrices from the matching cell types.

**Extended Data Fig. 10:**
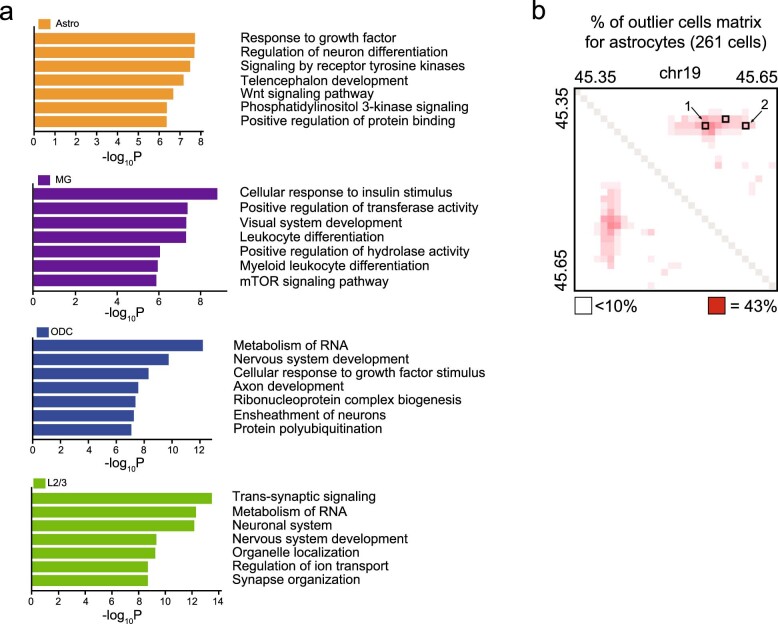
Application of SnapHiC to identify loops in specific cell types. **a**, Top seven enriched gene ontology (GO) terms of genes associated with Astro-specific, MG-specific, ODC-specific and L2/3-specific SnapHiC loops. The p-values are calculated based on the accumulative hypergeometric distribution. **b**, Matrix of the percentage of cells with significantly higher normalized contact frequency (percentage of outlier cells with normalized contact frequency>1.96) for 261 astrocytes around APOE gene. The two SnapHiC-identified loops from astrocyte are marked by black squares and their labels matched the numbers shown in Fig. 2c.

The accuracy and sensitivity of the above SnapHiC-identified loops were supported by two lines of evidence. First, APA analysis confirmed SnapHiC-identified loops show significant enrichment compared to their local background on the aggregated contact matrices (Extended Data Fig. 9). Second, anchors of SnapHiC-identified loops displayed corresponding cell type-specific chromatin accessibility, histone acetylation and gene expression in four distinct cell types: astrocytes, L2/3 excitatory neurons, oligodendrocytes and microglia, where assay for transposase-accessible chromatin using sequencing (ATAC-seq), H3K27ac ChIP–seq and RNA sequencing (RNA-seq) data are available^17,18^. To minimize the effect of cell number variation on the performance of SnapHiC, we randomly selected the same number of cells (*N* = 261) from astrocytes, oligodendrocytes and microglia to match the number of cells from L2/3 excitatory neurons, and applied SnapHiC to identify loops from these subsampled data. We found that most chromatin loops were cell type-specific (Supplementary Table 4), and the anchors of cell type-specific loops showed significantly higher ATAC-seq and H3K27ac ChIP–seq signals in the matched cell type compared to those in different cell types (Fig. 2b). The genes whose promoters linked to cell type-specific loops also showed significantly higher expression levels in the matched cell type (Fig. 2b and Supplementary Table 5). Moreover, they were associated with gene ontology terms^19^ related to cell type-specific biological processes (Extended Data Fig. 10a). Taken together, our results indicated that SnapHiC can detect chromatin loops reliably from scHi-C data in complex tissues.

Furthermore, we assigned candidate target genes to noncoding genome-wide association study (GWAS) single nucleotide polymorphisms (SNPs) based on the loops identified in specific cell types. We first collected 3,471 unique GWAS SNPs associated with seven neuropsychiatric disorders and traits that resided within the active enhancers of astrocytes, neurons, microglia or oligodendrocytes^17^ (Supplementary Table 6). Using SnapHiC-identified loops from the matching cell types (L2/3 excitatory neurons to represent neurons), we defined 788 SNP-gene linkages, connecting 445 disease-associated SNPs to 189 genes (Supplementary Table 7). The list included several known disease genes, such as *INPP5D* (Alzheimer’s disease), *RAB27B* (major depressive disorder, MDD), *SORL1* (Alzheimer’s disease) and *ZNF184* (MDD and schizophrenia). Figure 2c and Extended Data Fig. 10b showed an illustrative example of gene *APOE*, which was specifically expressed in astrocyte. Two astrocyte-specific loops connected the transcription start site of *APOE* to two active enhancers containing Alzheimer’s disease-associated GWAS SNPs (rs112481437 and rs138137383) in astrocyte. Our results indicated that *APOE* was the putative target gene of these two GWAS SNPs specifically in astrocytes.

In summary, we describe SnapHiC, a method to identify chromatin loops at high resolution and accuracy from sparse scHi-C datasets. Reanalyses of published scHi-C data from mES cells demonstrate that SnapHiC greatly boosts the statistical power in loop detection. Application of SnapHiC to sn-m3C-seq data from human prefrontal cortical cells reveals cell type-specific loops, which can predict putative target genes of noncoding GWAS SNPs. SnapHiC has the potential to facilitate the study of cell type-specific chromatin spatial organization in complex tissues.

## Methods

### Single-cell Hi-C (scHi-C) data processing

For scHi-C data from mES cells^1^, we downloaded the raw fastq files of all diploid serum cells. We first aligned scHi-C read pairs to mm10 genome with BWA-MEM with the ‘-5’ option, to report the most 5′ end alignment as the primary alignment, and the ‘-P’ option to perform the Smith–Waterman algorithm to rescue chimeric reads. We only used primary alignments in the next steps. We then deduplicated read pairs with the Picard tool to keep only one read pair at the exact same position. We further applied two filtering steps to remove duplications: (1) we split each chromosome into consecutive nonoverlapping 1-kb bins and only kept one contact for each 1-kb bin pair, and (2) we removed 1-kb bins that contact with more than ten other 1-kb bins, since they are likely mapping artifacts. The number of contacts per cell for all 1,175 cells has a bimodal distribution, and therefore only the top 742 cells with >150,000 contacts per cell were selected for downstream analysis.

### Single-nucleus methyl-3C-seq (sn-m3C-seq) data processing

For sn-m3C-seq data from human prefrontal cortex, we performed data processing using reference genome hg19 as described in the previous study^3^. Afterward, we applied the same filtering steps to remove duplications as described in the Single-cell Hi-C (scHi-C) data processing section. Again, the number of contacts per cell for all 4,238 cells showed a bimodal distribution and the top 2,869 cells with >150,000 contacts per cell were used for downstream analysis. The method for clustering and cell type annotation for these 2,869 cells was the same as previously described^3^.

### SnapHiC algorithm

#### Step A. Contact probability imputation using the RWR algorithm

We first partitioned each autosome into 10-kb bins and dichotomized contact for each 10-kb bin pair (binary contact matrix with 1 indicating nonzero contact and 0 otherwise). Next, we modeled each autosome as an unweighted graph, where each 10-kb bin is one node and each nonzero contact between any two 10-kb bins is one edge. We also added edges to all adjacent 10-kb bins. We then implemented the RWR algorithm^6^ with a restart probability of 0.05 to impute the contact probability between all intrachromosomal 10-kb bin pairs. We used the Python ‘NetworkX’ package to construct the graph and adopted the ‘linalg.solve’ function in the Python ‘SciPy’ package to solve the linear equation in the RWR algorithm. The systematic biases in imputed contact probabilities in scHi-C data are negligible, and thus normalization against effective fragment size, GC content or mappability is not needed (Supplementary Note).

#### Step B. Contact probability normalization based on one-dimensional (1D) genomic distance

Since the contact probability between any two genomic loci is dependent on their 1D genomic distance, normalization of the imputed contact probability against 1D genomic distance is needed before loop calling. To achieve this, we first removed bin pairs residing in the first 50 kb or the last 50 kb of each chromosome, which often have unusually high imputed contact probability due to the edge effect of the RWR algorithm. We then stratified all 10-kb bin pairs by their 1D genomic distance. Specifically, let *x*_*ij*_ represent the contact probability between bin *i* and bin *j*. Define set *A*_*d*_ as all bin pairs (*i*,*j*) with the 1D genomic distance *d*. For simplicity, we only considered bin pairs (*i*,*j*) in the upper triangle of the contact matrix where *i* < *j*. We removed the top 1% bin pairs in *A*_*d*_ with the highest contact probability, and then computed the mean *μ*_*d*_ and the standard deviation *σ*_*d*_ of the contact probability using the remaining bin pairs in *A*_*d*_. We further calculated the normalized contact probability (that is, *z* score), defined as *z*_*ij*_ = (*x*_*ij*_ − *μ*_*d*_)/*σ*_*d*_, for all bin pairs in *A*_*d*_. For single cells with very few contacts, the imputed contact probabilities *x*_*ij*_ at a specific 1D genomic distance *d* are close to zero, leading to very small standard deviation *σ*_*d*_ and numerical errors in the *z* score transformation. To avoid this issue, when *σ*_*d*_ is less than 10^−6^, we defined *z*_*ij*_ = 0 for all bin pairs in *A*_*d*_. After the calculation described above, bin pair (*i*,*j*) with higher normalized contact probability *z*_*ij*_ suggests that bin *i* and bin *j* are more likely to interact with each other than other loci pairs.

#### Step C. Identification of loop candidates

To minimize false positives in loop calling results, we defined a bin pair as a loop candidate only if it shows a higher contact probability compared to both its global and local background. Specifically, we required the loop candidate to satisfy the following criteria:

1. Its average normalized contact probability from all single cells is greater than 0 (that is, with respect to global background).
2. More than 10% of single cells have normalized contact probability above 1.96 (that is, *z* score >1.96, corresponding to a *z*-test two-sided *P* value <0.05, with respect to global background).
3. For each 10-kb bin pair (*i*,*j*), we defined its local neighborhood as all 10-kb bin pairs (*m*,*n*) such that 30 kb ≤ max{*d*(*i*,*m*), *d*(*j*,*n*)} ≤ 50 kb (Supplementary Fig. 1), where *d*(*i*,*m*) is the 1D genomic distance between the center of bin *i* and the center of bin *m*. Here we did not consider the bin pairs within 20 kb of bin pair (*i*,*j*) as part of its local neighborhood because they can be part of the same loop cluster centered at bin pair (*i*,*j*). We then compared the normalized contact probability at bin pair (*i*,*j*) with the mean of the normalized contact probability of all ninety-six 10-kb bin pairs within its local neighborhood region, and applied the paired *t*-test across all single cells to obtain a *P* value. We further converted *P* values into false discovery rates (FDRs) using the Benjamini–Hochberg procedure, again stratified by 1D genomic distance. A loop candidate must have FDR < 10% and *t*-statistics greater than three in the paired *t*-test (that is, with respect to local background).
4. Motivated by the HiCCUPS algorithm^4^, we also required each loop candidate to have at least 33% higher average normalized contact frequency than its circle, donut and lower left background and 20% higher average normalized contact frequency than its horizontal and vertical background (Supplementary Fig. 1) (that is, with respect to local background).
5. Finally, we removed loop candidates with either end having low mappability score (≤0.8), or overlapping with the ENCODE blacklist regions (http://mitra.stanford.edu/kundaje/akundaje/release/blacklists/mm10-mouse/mm10.blacklist.bed.gz for mm10 and https://www.encodeproject.org/files/ENCFF001TDO/ for hg19). The sequence mappability for each 10-kb bin is calculated based on our previous study^20^; it can be downloaded from http://enhancer.sdsc.edu/yunjiang/resources/genomic_features/.

#### Step D. Clustering of loop candidates and identifying the summit(s) as final outputs

For each loop candidate (*i*,*j*), we defined its surrounding area as all 10-kb bin pairs (*m*,*n*) such that max {*d*(*i*,*m*), *d*(*j*,*n*)} ≤ 20 kb, where *d*(*i*,*m*) is the 1D genomic distance between the center of bin *i* and the center of bin *m*. We defined a loop candidate as a singleton if there is no other loop candidate within its surrounding area, and removed all singletons from the downstream analysis since the singletons are likely to be false positives.

To group the remaining nonsingleton loop candidates into clusters, we adopted the Rodriguez and Laio’s algorithm^7^. Specifically, for each loop candidate (*i*,*j*), we first counted the number of loop candidates in its adjacent neighborhood regions: (*m*,*n*): max {*d*(*i*,*m*)},*d*(*j*,*n*) ≤ 10 kb, and defined this number as its local density *ρ*(*i*,*j*). Next, we calculated the minimum Euclidean distance between the loop candidate (*i*,*j*) and any other loop candidate with higher local density on the same chromosome, defined as *δ*(*i*,*j*):

$$\delta \left( {i,j} \right) = \mathop {{\min }}\limits_{\left( {m,n} \right):\rho \left( {m,n} \right) > \rho (i,j)} \sqrt {\left( {i - m} \right)^2 + \left( {j - n} \right)^2} .$$

If a loop candidate (*i*,*j*) has the highest local density (that is, *ρ*(*i*,*j*) = 9), *δ*(*i*,*j*) is defined as:

$$\delta \left( {i,j} \right) = \mathop {{\max }}\limits_{(m,n)} \sqrt {\left( {i - m} \right)^2 + \left( {j - n} \right)^2} .$$

We then selected loop candidates that have high local density *ρ*, and are relatively far away from other loop candidates with higher local density, that is, high *δ*, as loop cluster centers. To achieve this, let *ρ*_max_ and *δ*_max_ represent the maximal value of *ρ* and *δ* of all loop candidates on each chromosome, respectively. We defined $\rho ^{\prime} \left( {i,j} \right) = \rho \left( {i,j} \right)/\rho _{\mathrm{max}}$ and $\delta ^{\prime} \left( {i,j} \right) = \delta \left( {i,j} \right)/\delta _{\mathrm{max}}$ such that both $\rho ^{\prime} \left( {i,j} \right)$ and $\delta ^{\prime} \left( {i,j} \right)$ are within range [0,1]. We then defined $\eta \left( {i,j} \right) = \rho ^{\prime} \left( {i,j} \right) \times \delta ^{\prime} \left( {i,j} \right)$, ordered all loop candidates by their *η* in the descending order and plotted the rank of *η* against the value of *η*. In this plot, we selected the reflection point such that the slope at the reflection point is one. All loop candidates with *η* larger than *η* at the reflection point were chosen to be the loop cluster centers. After finding the loop cluster centers, we assigned each remaining loop candidate to the same loop cluster as its nearest neighbor with a higher local density *ρ*.

Within each loop cluster, we defined the loop candidate with the lowest FDR as the first summit of the cluster. For the first summit (*i*, *j*), we defined its surrounding area as all 10-kb bin pairs (*m*,*n*) such that max{*d*(*i*,*m*),*d*(*j*,*n*)} ≤ 20 kb, and removed all loop candidates within its surrounding area. Next, we selected the loop candidate with the lowest FDR among the remaining ones (if there is any) as the second summit of this cluster. We then removed all loop candidates within the surrounding area of the second summit in the same way as we did for the first summit, and searched for the third summit (if there is any) with the lowest FDR among the remaining loop candidates. Such a procedure was iterated until there were no loop candidates left in this cluster. Note that one loop cluster may contain multiple summits. The SnapHiC algorithm outputs a file containing the summit(s) of each loop cluster as its final chromatin loop list.

Details about the justification of the thresholds implemented in SnapHiC can be found in Supplementary Note and Supplementary Figs. 2 and 3.

### Identification of chromatin loops with SnapHiC

We applied SnapHiC to scHi-C data from 10, 25, 50, 75, 100, 200, 300, 400, 500, 600, 700 and 742 mES cells and each of the 14 cell clusters from sn-m3C-seq data of human prefrontal cortex to call chromatin loops at 10-kb resolution between the 100 kb and 1 Mb region on autosomal chromosomes.

We did not take bin pairs within 100 kb into consideration because they do not have complete information in their local neighborhood (refer to SnapHiC algorithm). We also evaluated the performance of SnapHiC beyond 1 Mb 1D genomic distance or at a different resolution; the results are summarized in the Supplementary Note.

### Visualization of scHi-C and sn-m3C-seq data using percentage (%) of outlier cells matrix

Due to the sparsity of the raw count matrix of scHi-C data, the SnapHiC-identified loops can be visualized by the percentage of the outlier cells matrix. Specifically, we first computed the percentage of outlier cells (that is, cells with normalized contact probability >1.96), and then took the integer ceiling of 100 × (% of outlier cells) to create a count matrix. We then used the Juicer^21^ software to convert the count matrix into a .hic file and visualize it in Juicebox^22^.

### Generation of aggregated contact matrix for scHi-C and sn-m3C-seq data

We pooled contacts from single cells of interest to create the aggregated contact matrix in .hic format using Juicer with KR normalization^21^. Only intrachromosomal contacts >2 kb away were used.

### Identification of loops/interactions using HiCCUPS, FastHiC, FitHiC2 and HiC-ACT from aggregated contact matrix

We applied the HiCCUPS^4^ to the aggregated contact matrix after pooling the contacts from single cells of interest and calling loops at 10-kb resolution with the two sets of parameters: (1) default parameter: ‘-ignore_sparsity -r 10000 -k KR -f.1 -p 2 -i 5 -t 0.02,1.5,1.75,2 -d 20000’ and (2) optimal parameter: ‘-ignore_sparsity -r 10000 -k KR -f .1 -p 4 -i 15 -t 0.4,1.5,1.75,2 -d 20000’.

We applied FitHiC2 (ref. ^5^), FastHiC^15^ and HiC-ACT^16^, with the default setting to the aggregated contact matrix after pooling the contacts from single cells of interest at 10-kb bin resolution. We also tested different significance thresholds to accommodate the sparse scHi-C data: FDR < 1%, <5% and <10% for FitHiC2; the posterior probability of significant interactions >0.9, >0.99 and >0.999 for FastHiC and the local neighborhood smoothed *P* values <10^−6^, <10^−^^7^ and <10^−^^8^ for HiC-ACT. After getting the raw output, we further removed significant chromatin interactions supported by fewer than six reads to minimize false positives. We then applied the same algorithm implemented in SnapHiC (Step D in SnapHiC algorithm) to identify their summits.

To ensure a fair comparison with SnapHiC-identified loops, we further filtered the above identified loops/interactions by selecting the intrachromosomal ones within 1D genomic distance roughly 100 kb–1 Mb and removing the loops whose anchor bins had low mappability (≤0.8) or overlapped with the ENCODE blacklist regions.

### Definition of loop overlap

Let bin pair (*i*,*j*) represent a loop in set *A*. We define that it overlaps with a loop in set *B*, if and only if there exists a loop (*m*,*n*) in set *B* such that max(*d*_*im*_, *d*_*jn*_) ≤ 20 kb, where *d*_*im*_ is the 1D genomic distance between the middle base pair of bin *i* and the middle base pair of bin *m*.

### Subsampling of scHi-C and sn-m3C-seq data

For scHi-C data from mES cells, we randomly permuted the order of all 742 cells that passing our quality control six times, and selected the first 10, 25, 50, 75, 100, 200, 300, 400, 500, 600 and 700 cells from all 742 cells to create a series of subsampled datasets.

For sc-m3C-seq data from human prefrontal cortex, we randomly permuted the order of all 338 astrocytes, 323 microglia and 1,038 oligodendrocytes and selected the first 261 cells to create the subsampled datasets for astrocytes, microglia and oligodendrocytes, respectively.

### Reproducibility of SnapHiC- and HiCCUPS-identified loops

Suppose we have two sets of loop list A and B. Let *P*_A_ represent the proportion of loops in set A overlapped with loops in set B (Definition of loop overlap) and let *P*_B_ represent the proportion of loops in set B overlapped with loops in set A. We used (*P*_A_ + *P*_B_)/2 to measure the reproducibility of loops in the two sets.

To assess the reproducibility of SnapHiC and HiCCUPS, we first randomly split all 742 mES cells into two groups where each group consists of 371 cells, and then applied SnapHiC and HiCCUPS to identify loops for each group. The reproducibility of SnapHiC- and HiCCUPS-identified loops between two sets of 371 cells was calculated as described above. We repeated such random splitting and loop calling analysis ten times, and reported the mean of reproducibility of SnapHiC and HiCCUPS-identified loops. We further used the paired *t*-test to evaluate the statistical significance of the difference in reproducibility between the methods.

### Generation of the reference loop/interaction lists for calculation of precision, recall and F1 score

For mES cells, the HiCCUPS loops at 10-kb resolution from bulk in situ Hi-C data were called as previously described^14^ using the pooled datasets of all four biological replicates from the Bonev et al. study^8^. MAPS^9^ was applied to H3K4me3 PLAC-seq data^9^, cohesin HiChIP data^10^ and H3K27ac HiChIP data^11^ to call significant intrachromosomal interactions at 10-kb resolution within 1 Mb. We combined the above four lists and further filtered by removing interactions where anchor bins had low mappability (≤0.8) or overlapped with the ENCODE blacklist regions to create the final reference loop list.

For oligodendrocytes, microglia and eight neuronal subtypes from human prefrontal cortex, we used MAPS-identified interactions from H3K4me3 PLAC-seq data of purified oligodendrocytes, microglia and neurons as their reference list, respectively^17^. We first filtered the list by selecting loops with 1D genomic distance roughly 100 kb–1 Mb and removing loops where anchor bins had low mappability (≤0.8) or overlapped with the ENCODE blacklist regions. We further selected the loops in which at least one end contains active promoters of the corresponding cell type to create the final reference interaction list.

### Calculation of precision, recall and F1 score

Let *N* represent the number of loops in the reference loop list for the cell type of interest. Suppose method A identifies *M* loops from the same cell type, and *m* of them overlapped with loops in the reference loop list (Definition of loop overlap). The precision is calculated as *m*/*M*. Suppose among all *N* loops in the reference loop list, *n* loops overlapped with method A-identified loops. The recall is calculated as *n*/*N*. Notably, *m* and *n* may not be equal since we allow up to a 20-kb gap between two overlapped loops. The F1 score is the harmonic mean of the precision and recall and is calculated as below:

$${\mathrm{F1}}\,{\mathrm{score}} = 2 \times \frac{{{\mathrm{Precision}} \times {\mathrm{Recall}}}}{{{\mathrm{Precision}} + {\mathrm{Recall}}}} = 2 \times \frac{{m/M \times n/N}}{{m/M + n/N}}.$$

For mES cells, we used all SnapHiC-, HiCCUPS-, FastHiC-, FitHiC2- or HiC-ACT-identified loops/interactions for the above calculation. For oligodendrocytes, microglia and eight neuronal subtypes, we only selected the SnapHiC-, HiCCUPS-, FastHiC-, FitHiC2- or HiC-ACT-identified loops/interactions in which at least one end contains active promoters of the corresponding cell type for this calculation, since the available reference loop lists are called from H3K4me3 PLAC-seq data, which can only detect interactions centered at promoter regions.

### APA

We used the Juicer^21^ software with the command ‘java -jar juicer_tools_1.19.02.jar apa -r 10000 -k KR -u input.hic loops.txt APA’ to perform the APA. We reported ‘P2LL’ (also known as the APA score) and ‘ZscoreLL’ to evaluate the enrichment of SnapHiC-identified loops with respect to the lower left background.

### CTCF motif orientation analysis

We obtained the CTCF ChIP–seq peaks of mES cells from Kubo et al.^23^ and used FIMO^24^ with default parameters and the CTCF motif (MA0139.1) from the JASPAR^25^ database to search for CTCF sequence motifs among CTCF ChIP–seq peaks. We then selected a subset of testable SnapHiC-identified loops in which both ends contain either a single CTCF motif or multiple CTCF motifs in the same direction and calculated the proportion of convergent, tandem and divergent CTCF motif pairs among all testable loops.

### Visualization of CTCF and H3K27ac ChIP–seq data from mES cells

We downloaded the signal tracks from the ENCODE portal (https://www.encodeproject.org/) with the following identifiers: ENCFF230RNU (for H3K27ac) and ENCFF069PTO (for CTCF) for Extended Data Fig. 4a.

### Definition of cell type-specific SnapHiC loops

We used the SnapHiC loops identified from subsampled astrocytes, microglia and oligodendrocytes datasets and L2/3 excitatory neurons (all with 261 cells) to define cell type-specific loops. We defined a loop identified from one cell type as cell type-specific, if it did not overlap (Definition of loop overlap) with loops identified from any of the other three cell types.

### Processing of ATAC-seq and H3K27ac ChIP–seq data from four brain cell types

The ATAC-seq and H3K27ac ChIP–seq data from human astrocytes, oligodendrocytes, microglia and neurons are from Nott et al.^17^ and are processed with ENCODE ATAC-seq and ChIP–seq pipelines as previously described^17^. The normalized bigwig tracks with reads per kilobase of a transcript, per million mapped reads as the *y* axis are generated for visualization in Fig. 2b.

### Processing of RNA-seq from four brain cell types

The RNA-seq data from human astrocytes, oligodendrocytes, microglia and neurons were acquired from Zhang et al.^18^. The alignment and quantification are performed with pipeline: https://github.com/ren-lab/rnaseq-pipeline. Briefly, we first aligned RNA-seq raw reads to hg19. Next, we used Gencode GTF gencode.v19.annotation.gtf for hg19 with STAR following the ‘ENCODE’ options outlined in the STAR manual (https://physiology.med.cornell.edu/faculty/skrabanek/lab/angsd/lecture_notes/STARmanual.pdf). We then used Picard (http://broadinstitute.github.io/picard/) to remove PCR duplicates. We also generated the normalized bigwig tracks with reads per kilobase of a transcript, per million mapped reads as the *y* axis for visualization in Fig. 2b.

### Enrichment analysis of ATAC-seq or H3K27ac ChIP–seq signals at cell type-specific loops

To quantify the intensity of ATAC-seq or H3K27ac ChIP–seq signals at cell type-specific loops in the cell type of interest, we first calculated reads per million (CPM) values in each 10-kb anchor of the cell type-specific loops using ATAC-seq or H3K27ac ChIP–seq data from the cell type of interest. To minimize the background noise, we only considered the reads falling into the ATAC-seq or H3K27ac ChIP–seq peak regions defined in the cell type of interest but not all the reads in the entire 10-kb bin. If there are multiple ATAC-seq or H3K27ac ChIP–seq peaks in the same 10-kb bin, we then added up the CPM values and took the sum as the value for that 10-kb bin. Since each loop has two anchors, we took their average CPM to represent the intensity of ATAC-seq or H3K27ac ChIP–seq signal for that loop in the cell type of interest. Last, we applied the paired Wilcoxon signed-rank test on log_2_(CPM+1) values from different combinations of cell types of interest and the cell type-specific loop sets to test whether there is a significant difference.

### Gene expression analysis at cell type-specific loops

We obtained the fragments per kilobase of transcript per million mapped reads (FPKM) values of each protein-coding gene in human astrocytes, neurons, microglia and oligodendrocytes from Supplementary Table 4 provided in Zhang et al. (Col P-U for astrocytes, Col AB for neurons, Col AC-AG for oligodendrocytes and Col AH-AJ for microglia in the ‘Human data only’ tab)^18^. For each gene, we took the average of FPKM across biological replicates of the same cell type. For the selected genes where promoters overlapped with cell type-specific loops, we applied the Wilcoxon signed-rank test to evaluate whether they were highly expressed in the matched cell type.

### Selection of GWAS SNPs associated with neuropsychiatric disorders and traits

We first collected 30,262 genome-wide significant (*P* < 5 × 10^−8^) noncoding GWAS SNPs associated with neuropsychiatric disorders and traits. We considered seven neuropsychiatric disorders, including Alzheimer’s disease^26^, attention deficit hyperactivity disorder^27^, autism spectrum disorder^28^, bipolar disorder^29^, intelligence quotient^30^, MDD^31^ and schizophrenia^32^, resulting in a total of 28,099 unique GWAS SNPs (Supplementary Table 6). We then overlapped these GWAS SNPs with active enhancers of astrocytes, neurons, microglia or oligodendrocytes defined in the previous study^17^ and this resulted in 3,639 SNP-disease associations (3,471 unique GWAS SNPs) for analysis (Supplementary Table 6).

### Reporting Summary

Further information on research design is available in the Nature Research Reporting Summary linked to this article.

## Online content

Any methods, additional references, Nature Research reporting summaries, source data, extended data, supplementary information, acknowledgements, peer review information; details of author contributions and competing interests; and statements of data and code availability are available at 10.1038/s41592-021-01231-2.

## Supplementary information

Supplementary Information Supplementary Figs. 1–4 and Note.

Reporting Summary

Supplementary Tables 1–7.

## Data Availability

The scHi-C data from mES cells were downloaded from https://www.ncbi.nlm.nih.gov/geo/query/acc.cgi?acc=GSE94489. The sn-m3C-seq data from human prefrontal cortex were downloaded from https://www.ncbi.nlm.nih.gov/geo/query/acc.cgi?acc=GSE130711. The ATAC-seq and H3K27ac ChIP–seq data from human astrocytes, oligodendrocytes, microglia and neurons were downloaded from dbGap (https://www.ncbi.nlm.nih.gov/projects/gap/cgi-bin/study.cgi?study_id=phs001373.v2.p2). The RNA-seq data from human astrocytes, oligodendrocytes, microglia and neurons were downloaded from https://www.ncbi.nlm.nih.gov/geo/query/acc.cgi?acc=GSE73721. The signal tracks of CTCF and H3K27ac ChIP–seq data for mES cells (Extended Data Fig. 4a) were downloaded from the ENCODE portal (https://www.encodeproject.org/) with the following identifiers: ENCFF230RNU (for H3K27ac) and ENCFF069PTO (for CTCF). The hg19 and mm10 reference genomes were downloaded from https://hgdownload.soe.ucsc.edu/goldenPath/hg19/bigZips/hg19.fa.gz and https://hgdownload.soe.ucsc.edu/goldenPath/mm10/bigZips/mm10.fa.gz, respectively. The full lists of interactions/loops identified by different methods are provided as source data. Source data are provided with this paper.

## Source data

Source Data Fig. 1 SnapHiC-identified loops from different numbers of mES cells (*N* = 10, 25, 50, 75, 100, 200, 300, 400, 500, 600, 700, 742). For *N* = 10, 25, 50, 75, 100, 200, 300, 400, 500, 600, 700, loop calling was repeated six times on the subsampling datasets randomly selected from the 742 cells that passed our quality control. The tab name indicates the subsampling index (that is, ‘Permu0’, ‘Permu1’, ‘Permu2’, ‘Permu3’, ‘Permu4’, ‘Permu5’) and the number of cells used (that is, ‘10’, ‘25’, ‘50’, ‘75’, ‘100’, ‘200’, ‘300’, ‘400’, ‘500’, ‘600’, ‘700’, ‘742’).
Source Data Fig. 2 SnapHiC-identified loops from astrocytes, microglia and oligodendrocytes after subsampling (261 cells for each cell type) and the cell-type-specific loops from astrocytes, microglia, oligodendrocytes and L2/3 excitatory neurons after subsampling.
Source Data Extended Data Fig. 2 HiCCUPS-identified loops from bulk in situ Hi-C data, MAPS-identified significant interactions from H3K4me3 PLAC-seq, cohesin HiChIP and H3K27ac HiChIP data from mES cells, which are used as the reference list after pooling to calculate precision rate, recall rate and the F1 score for loops/interactions identified from mES cells.
Source Data Extended Data Fig. 7 SnapHiC-identified loops from each of the 14 different cell types in human prefrontal cortex. The tab name indicates the cell type.
Source Data Extended Data Fig. 8 MAPS-identified interaction lists of human microglia (MG), oligodendrocytes (ODC) and neurons from the Nott. et al. study, which are used to calculate precision rate, recall rate and the F1 score for MG, ODC and eight neuronal subtypes (L2/3, L4, L5, L6, Sst, Vip, Ndnf and Pvalb), respectively.

## Code availability

The SnapHiC software package with a detailed user tutorial and sample input and output files can be found at https://github.com/HuMingLab/SnapHiC.
