# Supplementary material for: SnapHiC: a computational pipeline to identify chromatin loops from single-cell Hi-C data
Source: Nat Methods. 2021 Aug 26;18(9):1056–9. doi: 10.1038/s41592-021-01231-2 (PMC8440170; doi:10.1038/s41592-021-01231-2)
Supplement: Supplementary file 1 — Supplementary Figs. 1–4 and Note. [file 41592_2021_1231_MOESM1_ESM.pdf]

nature portfolio

https://doi.org/10.1038/s41592-021-01231-2

Supplementary information

SnapHiC: a computational pipeline to identify chromatin loops from single-cell Hi-C data

In the format provided by the authors and unedited

Supplementary information

Supplementary Figure

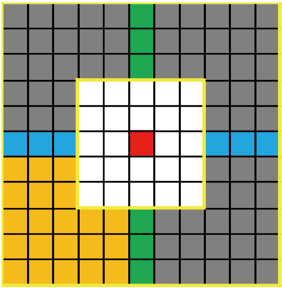

Supplementary Figure 1. Illustration of different types of the local background used for SnapHiC loop calling. For each 10Kb bin pair of interest (red), its horizontal background, vertical background, lower left background and donut background are the blue, green, yellow and grey areas, respectively. The circle background, which is also the local neighborhood, is the union of the blue, green, yellow and grey areas.

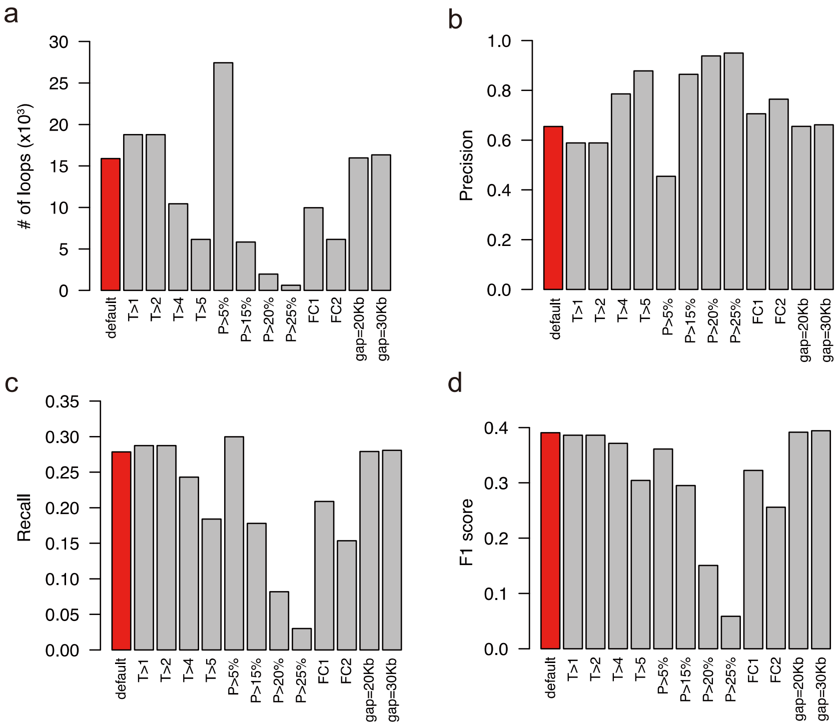

Supplementary Figure 2. Performance of SnapHiC with different parameter configurations on 742 mES cells. The number of identified loops (**a**), precision (**b**), recall (**c**) and F1 score (**d**).

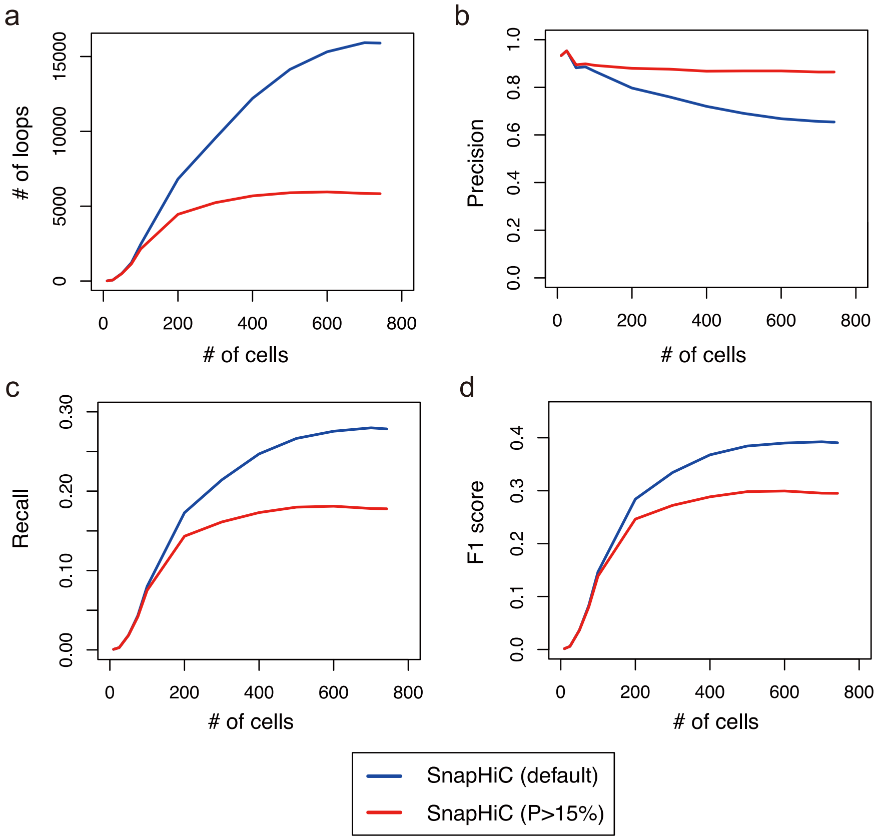

Supplementary Figure 3. Comparison of the performance of SnapHiC with default parameter and P>15% on different numbers of mES cells. Line plots showing the number of identified loops (**a**), precision (**b**), recall (**c**) and F1 score (**d**) on different number of mES cells (N=10, 25, 50, 75, 100, 200, 300, 400, 500, 600, 700 and 742).

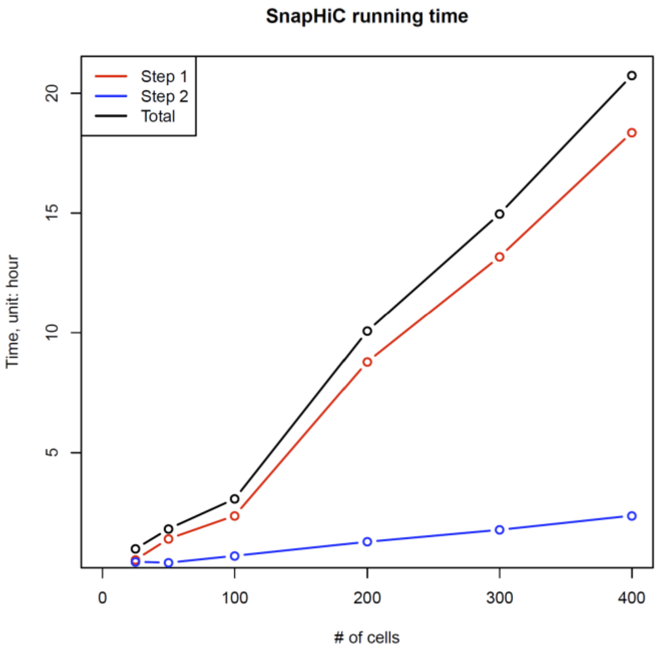

Supplementary Figure 4. The relationship between the number of cells and the running time of SnapHiC analysis. We tested the running time of SnapHiC on scHi-C data from 25, 50, 100, 200, 300 and 400 mES cells (10Kb resolution, searching for loops between 100Kb and 1Mb 1D genomic distance). SnapHiC consists of two steps: (1) applying the random walk with restart (RWR) algorithm to impute contact probability within every single cell, and (2) integrating imputed contact probability matrices from all single cells to identify chromatin loops. The running time of each step and the sum of both steps against the number of cells is plotted.

Supplementary note

Optimization of HiCCUPS parameters for loop calling from aggregated single cell Hi-C (scHi-C) data

As recommended in Rao et al. 2014 study[1], The default parameter of HiCCUPS running at 10Kb resolution is “-f .1 -p 2 -i 5 -t 0.02,1.5,1.75,2 -d 20000”. Since the default parameters are tuned using deeply sequenced bulk Hi-C data with over billions of raw reads, applying HiCCUPS with default parameter finds very few loops on the aggregated scHi-C, resulting in low recall and low F1 score (**Fig. 1b, 1c**).

To improve the performance of HiCCUPS on sparse data, we examined how changes of each parameter may affect the number of loops identified from the aggregated scHi-C data of the 742 mES cells. Specifically, the following parameters are considered:

1) The window width used for finding enriched pixels (-i) from 1 to 4;
2) Peak width used for finding enriched pixels (-p) from 3 to 15;
3) Threshold allowed for the sum of FDR values of the horizontal, vertical, donut, and bottom left filters (1st value in -t) from 0.02 to 0.4;
4) The threshold ratios for three types of local background (2nd to 4th values in -t) of “1.5,1.75,2” and “1.2,1.33,1.33”.

Notably, we did not change the default false discovery rate threshold 10% (-f), since FDR<10% is already lenient and has been widely used; we also did not change the default distance 20Kb (-d) to merge nearby pixels, since SnapHiC also applied the same 20Kb distance for merging. We only made change to one parameter each time for test, and kept the other parameters as default values.

We found that for most parameters mentioned above, applying HiCCUPS with more lenient thresholds results in more loops with a higher F1 score compared to the default parameters (**Extended Data Fig. 1**). The only exception is the threshold ratios for three types of local background: using “1.2,1.33,1.33” instead of the default “1.5,1.75,2” identified only ~1% more loops. To maximize the number of identified loops on aggregated scHi-C data, we chose the most lenient thresholds of each parameter and termed such combination as the “optimal” parameter (“-f .1 -p 4 -i 15 -t 0.4,1.5,1.75,2 -d 20000”) for comparison with SnapHiC.

Comparison of SnapHiC with additional computation tools designed for bulk Hi-C to identify chromatin interactions

In addition to HiCCUPS which identifies chromatin loops based on the local background model, many methods have been developed to identify significant chromatin interactions based on the global background model. Therefore, we selected three representative methods, FastHiC[2], FitHiC2[3] and HiC-ACT[4], and compared them with SnapHiC. FastHiC is a hidden Markov random field (HMRF) based Bayesian method which can explicitly model the spatial dependency of chromatin interactions among adjacent bin pairs. FastHiC used posterior probability to determine the statistical significance of chromatin interactions. FitHiC2, the latest reimplementation of Fit-Hi-C[5], first fitted a non-parametric spline to estimate the 1D genomic distance effect of Hi-C contact frequency, and then used p-value from Binomial distribution and the corresponding false discovery rate (FDR) to determinate the statistical significance of chromatin interactions. HiC-ACT is an aggregated Cauchy test (ACT)-based approach to

improve the accuracy of chromatin interactions by post-processing the results from other methods. HiC-ACT used the local neighborhood smoothed p-value to determine the statistical significance of chromatin interactions.

These three methods were selected because: (1) FastHiC has the best performance with down-sampled bulk Hi-C data as shown in Li et al. study[6]; (2) Based on the results from previous review paper that compare the performance of different computational methods for Hi-C data[7], Fit-Hi-C performs well in interaction identification and it is also one of the most used methods; FitHiC2 is its improved version; (3) HiC-ACT is a recently developed method to identify significant chromatin interactions from bulk Hi-C data and it has been shown to achieve improved sensitivity with controlled type I error.

It is notable that, unlike HiCCUPS or SnapHiC, these three methods use the global background model and treat nearby significant chromatin interactions as independent units. Therefore, to make a fair comparison between the results from these three methods and those from SnapHiC and HiCCUPS, we only selected summits from their default output for our analysis (see details in **Methods**). Since these methods are designed for bulk Hi-C and their default significance thresholds may not be optimized for single cell Hi-C data, we also tested different significance thresholds for each method.

Justification of threshold values used in the SnapHiC

The current default parameters recommended in SnapHiC were optimized using mES cells, because it has a rich set of deeply sequenced Hi-C, PLAC-seq and HiChIP datasets[8-11], allowing us to empirically evaluate the performance of SnapHiC with different parameters. When we optimized SnapHiC, the goal is to achieve an overall high F1 score across different cell numbers, especially when the cell number is low. In addition, we also aim to have a minimum precision of ~65%, considering the reproducibility of HiCCUPS loops between deeply sequenced biological replicates is about this level (Fig. 3B in Rao et al. 2014 study[1]).

Below is a list of key threshold values that affect the performance of SnapHiC, and the alternative values. To demonstrate the effect of each parameter, we only made change to one parameter each time, and kept the other parameters as the default values.

(1) Paired T-test statistics. The default is T>3. We evaluated four alternatives: T>1, T>2, T>4 and T>5.

(2) Proportion of outlie cells (i.e., cells with Z-score>1.96). The default is P>10%. We evaluated four alternatives: P>5%, P>15%, P>20% and P>25%.

(3) Fold change enrichment over five types of local background models.

Default: Circle>1.33, Donut>1.33, lower left>1.33, horizontal>1.2, vertical>1.2

We evaluated two alternatives with more stringent threshold values:

FC1: Circle>1.38, Donut>1.38, lower left>1.38, horizontal>1.25, vertical>1.25

FC2: Circle>1.43, Donut>1.43, lower left>1.43, horizontal>1.3, vertical>1.3

(4) Merging nearby loop candidates into loop clusters. The default gap in merging is 10Kb. We evaluated two alternatives: gap = 20Kb and gap = 30Kb.

Supplementary Fig. 2 shows the number of loops, precision, recall and F1 score for each parameter configuration on 742 mES cells. The default SnapHiC parameters achieved a good balance between precision and recall with the highest F1 score. If more conservative loops are preferred, users may change the proportion of outlier cells from >10% to >15%. From test results on different number of mES cells**,** such change yields a lower F1 score but increased precision (**Supplementary Fig. 3**)**.**

Evaluation of systematic biases for contact probability imputed by the RWR algorithm

We further evaluated whether the contact probability imputed by the RWR algorithm in each single cell contains systematic biases, including effective fragment size, GC content and mappability, which are known systematic biases in bulk Hi-C data[12]. Specifically, for each of the 742 mES scHi-C profiles, we used the RWR algorithm to impute the contact probability between all intra-chromosomal 10Kb bin pairs (i,j) within 1Mb at 1D genomic distance, denoted as x_{ij}. Let F_i, GC_i and M_i represent the effective fragment size, GC content and mappability of the 10Kb bin i, which are calculated according to our previous work[12]. We define $f_{ij} = F_i * F_j$, $gc_{ij} = GC_i * GC_j$, and $m_{ij} = M_i * M_j$, as the measure of three types of bias for each 10Kb bin pair. We then calculated the Pearson Correlation Coefficient between the contact probability x_{ij} and f_{ij}, gc_{ij} and m_{ij}, respectively, for each of the 19 autosomal chromosomes in one cell. Next, we used the average Pearson Correlation Coefficient (aPCC) across all chromosomes as the measurement of bias in each cell. Among all 742 cells, the mean of aPCC is 0.0110, 0.0085 and -0.0016 for effective fragment size, GC content and mappability, respectively. The standard deviation of aPCC is 0.0068, 0.0113 and 0.0029 for effective fragment size, GC content and

mappability, respectively. These results suggest that the systematic biases in imputed contact probabilities in scHi-C data are negligible, thus normalization against effective fragment size, GC content or mappability is not needed.

Computational cost (memory, time) of SnapHiC

To assess the relationship between the number of cells and running time, we tested the running time of SnapHiC on 25, 50, 100, 200, 300 and 400 mES cells (10Kb resolution, searching for loops between 100Kb to 1Mb) and found its running time increases linearly with the increase of cell number (**Supplementary Fig. 4**).

As described in our GitHub website (https://github.com/HuMingLab/SnapHiC), SnapHiC consists of two steps: (1) applying the random walk with restart (RWR) algorithm to impute contact probability within each single cell, and (2) integrating imputed contact probability matrices from all single cells to identify significant chromatin loops. Since the RWR algorithm can be applied to each chromosome in each single cell in parallel, in step 1, using as many processors as possible (e.g., maximal N = # of cells * # of chromosomes) can speed up the computation. Resolution and chromosome size are two important factors to determine the required memory per processor in step 1. For human or mouse genome at 10Kb resolution, we recommend allocating at least 30GB of memory for each processor. In the benchmarking experiments shown in **Supplementary Fig. 4**, we used 45 processors (15 nodes, 3 processors per node) for step 1, where each node has 96GB of memory, and it takes around 2.4 hours to process 100 cells.

In step 2, since the computation is performed jointly for all cells and separately for each chromosome, we recommend using the same number of processors as the number of chromosomes. Using more processors than that will be a waste of computing resources. It is also important to ensure that each processor has access to sufficient memory for the computation over all cells, and the amount of memory needed is correlated with the range of 1D genomic distance, the bin resolution, and to a less extent to the number of cells. Increasing the number of cells, slightly adds to the memory usage, however, since we only load the indices in the matrix that are used in each step of the computation, this increase in memory usage is sublinear in regard to the increase in the number of cells. In the benchmarking experiments shown in **Supplementary Fig. 4**, we used 20 processors (5 nodes, 4 processors per node) for

step 2, where each node has 96GB of memory, and it takes around 0.7 hours to process 100 cells in step 2.

The performance of SnapHiC beyond 1Mb at 1D genomic distance or at a different resolution

When we extended the maximal 1D genomic distance from 1Mb to 2Mb for loop calling on scHi-C data from 742 mES cells, only 4.6% SnapHiC-identified loops (758 out of 16,654) are between 1Mb and 2Mb. Therefore, we restricted our loop calling from 100Kb to 1Mb for all the datasets mentioned in this study. In practice, we also suggest using 1Mb as the maximal 1D genomic distance for loop calling to save computational cost.

We also tested the performance of SnapHiC of calling chromatin loops at 20Kb resolution on 742 mES cells. At 20Kb resolution, SnapHiC identified 17,078 loops, with 63.8% precision, 35.9% recall and F1-score of 0.460. These results are comparable with those from the default 10Kb resolution (15,896 loops with 65.5% precision, 27.8% recall and F1-score of 0.391). In addition, 8,280 out of 17,078 (48.5%) 20Kb loops overlapped with 10Kb loops. Given the robust performance of SnapHiC at both 10Kb and 20Kb resolutions, the SnapHiC pipeline allows users to choose from two different bin sizes (10Kb and 20Kb) with 10Kb as the default (https://github.com/HuMingLab/SnapHiC/tree/master/ext).

Use of single cell 3D structures to identify chromatin loops.

Recent studies[13-15] have shown that computational methods can predict full 3D genome structures in every single cell, providing another promising way to identify chromatin loops. Without relying on the random walk with restart imputation, one may use the 3D coordinates of each genomic locus in the predicted 3D model to calculate the Euclidean distance between any loci pairs, and define loci pairs with close spatial proximity as chromatin loops. However, besides the requirement of sufficient unique contacts per cell, haplotype information is also needed for 3D modeling based on previous studies[13-15], which is usually hard, if not impossible, to obtain. Moreover, it is still not clear exactly how the 3D model information should be used to infer high-resolution chromatin loops, an aspect that needs further exploration in the future.

Use of other scHi-C preprocessing methods

When our manuscript was under revision, three scHi-C data preprocessing methods, Higashi[16], BandNorm and 3DVI[17], were posted in bioRxiv. Although these methods achieved promising

results for mapping large-scale chromatin organization features, such as the identification of A/B compartments and TAD-like structures at 50Kb resolution, their performance on normalization and imputation of scHi-C data at 10Kb resolution has not been fully evaluated. In addition, Higashi, BandNorm and 3DVI are still under active development, and may have major updates in the near future. Therefore, in this study, we only used the RWR[18] algorithm to impute the contact probability at 10Kb bin resolution within each cell. Considering the rapid advances in the scHi-C data preprocessing methods, SnapHiC can also take the imputed contact matrices generated by methods other than the RWR algorithm as input. Future studies are needed to benchmark the performance of different scHi-C preprocessing methods, and evaluate their impacts on loop detection.

References

1 Rao, Suhas S. P. *et al.* A 3D Map of the Human Genome at Kilobase Resolution Reveals Principles of Chromatin Looping. *Cell* **159**, 1665-1680 (2014).

2 Xu, Z., Zhang, G., Wu, C., Li, Y. & Hu, M. FastHiC: a fast and accurate algorithm to detect long-range chromosomal interactions from Hi-C data. *Bioinformatics (Oxford, England)* **32**, 2692-2695 (2016).

3 Kaul, A., Bhattacharyya, S. & Ay, F. Identifying statistically significant chromatin contacts from Hi-C data with FitHiC2. *Nature protocols* **15**, 991-1012, doi:10.1038/s41596-019-0273-0 (2020).

4 Lagler, T. M., Abnousi, A., Hu, M., Yang, Y. & Li, Y. HiC-ACT: improved detection of chromatin interactions from Hi-C data via aggregated Cauchy test. *American journal of human genetics* **108**, 257-268, doi:10.1016/j.ajhg.2021.01.009 (2021).

5 Ay, F., Bailey, T. L. & Noble, W. S. Statistical confidence estimation for Hi-C data reveals regulatory chromatin contacts. *Genome Res* **24**, 999-1011, doi:10.1101/gr.160374.113 (2014).

6 Li, X., An, Z. & Zhang, Z. Comparison of computational methods for 3D genome analysis at single-cell Hi-C level. *Methods*, doi:10.1016/j.ymeth.2019.08.005 (2019).

7 Forcato, M. *et al.* Comparison of computational methods for Hi-C data analysis. *Nature methods* **14**, 679-685, doi:10.1038/nmeth.4325 (2017).

8 Bonev, B. *et al.* Multiscale 3D Genome Rewiring during Mouse Neural Development. *Cell* **171**, 557-572.e524, doi:10.1016/j.cell.2017.09.043 (2017).

9 Juric, I. *et al.* MAPS: model-based analysis of long-range chromatin interactions from PLAC-seq and HiChIP experiments. *PLoS computational biology* **In press.**, doi:10.1101/411835 (2019).

10 Mumbach, M. R. *et al.* HiChIP: efficient and sensitive analysis of protein-directed genome architecture. *Nature methods* **13**, 919-922, doi:10.1038/nmeth.3999 (2016).

11 Mumbach, M. R. *et al.* Enhancer connectome in primary human cells identifies target genes of disease-associated DNA elements. *Nature genetics* **49**, 1602-1612, doi:10.1038/ng.3963 (2017).

12 Hu, M. *et al.* HiCNorm: removing biases in Hi-C data via Poisson regression. *Bioinformatics (Oxford, England)* **28**, 3131-3133, doi:10.1093/bioinformatics/bts570 (2012).

13 Tan, L., Xing, D., Chang, C. H., Li, H. & Xie, X. S. Three-dimensional genome structures of single diploid human cells. *Science (New York, N.Y.)* **361**, 924-928, doi:10.1126/science.aat5641 (2018).

14 Tan, L., Xing, D., Daley, N. & Xie, X. S. Three-dimensional genome structures of single sensory neurons in mouse visual and olfactory systems. *Nature structural & molecular biology* **26**, 297-307, doi:10.1038/s41594-019-0205-2 (2019).

15 Tan, L. *et al.* Changes in genome architecture and transcriptional dynamics progress independently of sensory experience during post-natal brain development. *Cell* **184**, 741-758.e717, doi:10.1016/j.cell.2020.12.032 (2021).

16 Zhang, R., Zhou, T. & Ma, J. Multiscale and integrative single-cell Hi-C analysis with Higashi. 2020.2012.2013.422537, doi:10.1101/2020.12.13.422537 %J bioRxiv (2021).

17 Zheng, Y., Shen, S. & Keleş, S. Normalization and De-noising of Single-cell Hi-C Data with BandNorm and 3DVI. 2021.2003.2010.434870, doi:10.1101/2021.03.10.434870 %J bioRxiv (2021).

18 Zhou, J. *et al.* Robust single-cell Hi-C clustering by convolution- and random-walk-based imputation. *Proceedings of the National Academy of Sciences of the United States of America* **116**, 14011-14018, doi:10.1073/pnas.1901423116 (2019).
