# Supplementary material for: SnapHiC: a computational pipeline to identify chromatin loops from single-cell Hi-C data
Source: Nat Methods. 2021 Aug 26;18(9):1056–9. doi: 10.1038/s41592-021-01231-2 (PMC8440170; doi:10.1038/s41592-021-01231-2)
Supplement: Supplementary file 2 — Reporting Summary [file 41592_2021_1231_MOESM2_ESM.pdf]

nature research

Corresponding author(s): Ming Hu, Bing Ren

Last updated by author(s): 06/11/2021

Reporting Summary

Nature Research wishes to improve the reproducibility of the work that we publish. This form provides structure for consistency and transparency in reporting. For further information on Nature Research policies, see our Editorial Policies and the Editorial Policy Checklist.

Statistics

For all statistical analyses, confirm that the following items are present in the figure legend, table legend, main text, or Methods section.

n/a	Confirmed	
☐	☒	The exact sample size (*n*) for each experimental group/condition, given as a discrete number and unit of measurement
☐	☒	A statement on whether measurements were taken from distinct samples or whether the same sample was measured repeatedly
☐	☒	The statistical test(s) used AND whether they are one- or two-sided<br>*Only common tests should be described solely by name; describe more complex techniques in the Methods section.*
☐	☒	A description of all covariates tested
☐	☒	A description of any assumptions or corrections, such as tests of normality and adjustment for multiple comparisons
☐	☒	A full description of the statistical parameters including central tendency (e.g. means) or other basic estimates (e.g. regression coefficient) AND variation (e.g. standard deviation) or associated estimates of uncertainty (e.g. confidence intervals)
☐	☒	For null hypothesis testing, the test statistic (e.g. F, t, r) with confidence intervals, effect sizes, degrees of freedom and P value noted<br>*Give P values as exact values whenever suitable.*
☒	☐	For Bayesian analysis, information on the choice of priors and Markov chain Monte Carlo settings
☒	☐	For hierarchical and complex designs, identification of the appropriate level for tests and full reporting of outcomes
☐	☒	Estimates of effect sizes (e.g. Cohen's d, Pearson's r), indicating how they were calculated

Our web collection on statistics for biologists contains articles on many of the points above.

Software and code

Policy information about availability of computer code

Data collection	No software was used for data collection.
Data analysis	We used R version 3.6.1, Python version 3.6, Java version 8, Juicer version 1.6, Juicebox version 1.11.08, MAPS version 1.0.0, FitHiC version 2.0, FastHiC version 1.0, HiC-ACT version 021121, FIMO version 5.1.1, STAR version 2.3.0 and Metascape version 3.5 (https://metascape.org/gp/index.html#/main/step1), in the data analysis. SnapHiC version 0.1.0 code is available at https://github.com/HuMingLab/snapHiC.

For manuscripts utilizing custom algorithms or software that are central to the research but not yet described in published literature, software must be made available to editors and reviewers. We strongly encourage code deposition in a community repository (e.g. GitHub). See the Nature Research guidelines for submitting code & software for further information.

Data

Policy information about availability of data

All manuscripts must include a data availability statement. This statement should provide the following information, where applicable:

- Accession codes, unique identifiers, or web links for publicly available datasets
- A list of figures that have associated raw data
- A description of any restrictions on data availability

The scHi-C data from mES cells was downloaded from https://www.ncbi.nlm.nih.gov/geo/query/acc.cgi?acc=GSE94489. The sn-m3C-seq data from human prefrontal cortex was downloaded from https://www.ncbi.nlm.nih.gov/geo/query/acc.cgi?acc=GSE130711. The ATAC-seq and H3K27ac ChIP-seq data from human astrocytes, oligodendrocytes, microglia and neurons was downloaded from dbGap (https://www.ncbi.nlm.nih.gov/projects/gap/cgi-bin/study.cgi?study_id=phs001373.v2.p2). The RNA-seq data from human astrocytes, oligodendrocytes, microglia and neurons was downloaded from https://www.ncbi.nlm.nih.gov/geo/query/acc.cgi?acc=GSE73721. The signal tracks of CTCF and H3K27ac ChIP-seq data for mES cells (Extended Data Fig. 4a) were downloaded from the ENCODE portal (https://www.encodeproject.org/) with the following identifiers: ENCFF230RNU (for H3K27ac) and ENCFF069PTO (for CTCF).

The hg19 and mm19 reference genomes were downloaded from https://hgdownload.soe.ucsc.edu/goldenPath/hg19/bigZips/hg19.fa.gz and https://hgdownload.soe.ucsc.edu/goldenPath/mm10/bigZips/mm10.fa.gz, respectively. The full lists of interactions/loops identified by different methods are provided as supplementary source data. Source data are provided with this paper.

Field-specific reporting

Please select the one below that is the best fit for your research. If you are not sure, read the appropriate sections before making your selection.

☒ Life sciences ☐ Behavioural & social sciences ☐ Ecological, evolutionary & environmental sciences

For a reference copy of the document with all sections, see nature.com/documents/nr-reporting-summary-flat.pdf

Life sciences study design

All studies must disclose on these points even when the disclosure is negative.

Sample size	The performance of SnapHiC was tested on single cell Hi-C data from mES cells and 14 different cell types from human brain, which was sufficient to demonstrate the robustness of SnapHiC algorithm.
Data exclusions	We observed a bi-modal distribution of contacts for each cell in both Nagano et al. 2017 and Lee et al. 2019 studies. We removed cells with low contacts (<150,000) per cell, and only kept cells with high contacts (>=150,000) per cell for single cell Hi-C loop calling analysis. Such data exclusion criterion is pre-established before downstream analysis.
Replication	Our study focus on single cell Hi-C data analysis and each cell can only be measured once.
Randomization	When applying our SnapHiC method on different number of single cells, we randomly selected the cells to be analyzed.
Blinding	Since we used the publicly available single cell Hi-C data generated by Nagano et al. 2017 study (PMID: 28682332, GSE94489) and Lee et al. 2019 study (PMID: 31501549, GSE130711), blinding was not relavant to our study.

Reporting for specific materials, systems and methods

We require information from authors about some types of materials, experimental systems and methods used in many studies. Here, indicate whether each material, system or method listed is relevant to your study. If you are not sure if a list item applies to your research, read the appropriate section before selecting a response.

Materials & experimental systems

n/a	Involved in the study	
☒	☐	Antibodies
☒	☐	Eukaryotic cell lines
☒	☐	Palaeontology and archaeology
☒	☐	Animals and other organisms
☒	☐	Human research participants
☒	☐	Clinical data
☒	☐	Dual use research of concern

Methods

n/a	Involved in the study	
☒	☐	ChIP-seq
☒	☐	Flow cytometry
☒	☐	MRI-based neuroimaging
